# Patients admitted to hospital after suicide attempt with violent methods compared to patients with deliberate self-poisoning -a study of background variables, somatic and psychiatric health and suicidal behavior

**DOI:** 10.1186/s12888-018-1602-5

**Published:** 2018-01-24

**Authors:** Per Sverre Persett, Tine K. Grimholt, Oivind Ekeberg, Dag Jacobsen, Hilde Myhren

**Affiliations:** 1 Department of Acute Medicine, Oslo University Hospital, Oslo, Norway; 2 Divisions of Mental Health and Addiction, Oslo, Norway; 3 Regional Centers of Violence, Traumatic Stress and Suicide Prevention Eastern Norway, Oslo, Norway

**Keywords:** Emergency room, Injury and severity score, Deliberate self-poisoning, Suicide attempt and violent methods

## Abstract

**Background:**

In Norway, there are about 550 suicides recorded each year. The number of suicide attempts is 10–15 times higher. Suicide attempt is a major risk factor for suicide, in particular when violent methods are used. Suicide attempts with violent methods have hardly been studied in Norway. This study describes demographic, psychiatric and somatic health in patients admitted to somatic hospitals in Norway after suicide attempt by violent methods compared with suicide attempters using deliberate self-poisoning (DSP).

**Methods:**

Patients admitted to somatic hospital after suicide attempt aged > 18 years were included in a prospective cohort study, enrolled from December 2010 to April 2015.

Demographics (gender, age, marital and living condition, educational and employment status), previous somatic and psychological health were registered. Patients who had used violent methods were compared with patients admitted after suicide attempt by DSP.

**Results:**

The study included 80 patients with violent methods and 81 patients with DSP (mean age both groups 42 yrs.). Violent methods used were cutting (34%), jumping from heights (32%), hanging (14%), others (10%), shooting (7%) and drowning (4%).

Patients with violent methods had more often psychosis than patients admitted with DSP (14% vs 4%, *p* <  0.05), less anxiety disorders (4% vs 19%, *p* <  0.01) and less affective disorders (21% vs. 36%, *p* <  0.05). There were no significant differences between the numbers of patients who received psychiatric treatment at the time of the suicide attempt (violent 55% versus DSP 48%) or reported previous suicide attempt, 58% in patients with violent methods and 47% in DSP. Patients with violent methods stayed longer in hospital (14.3 (mean 8.3–20.3) vs. 2.3 (mean 1.6–3.1) days, *p* <  0.001), stayed longer in intensive care unit (5 days vs. 0.5 days, *p* <  0.001) and were in need of longer mechanical ventilation (1.4 vs 0.1 days, *p* <  0.001).

**Conclusions:**

Patients with violent methods had more often psychosis, less anxiety disorders and affective disorders than patients with DSP. Psychiatric treatment before the attempt and previous suicide attempt was not significantly different between the groups and about half of the patients in both groups were in psychiatric treatment at the time of the suicide attempt.

## Background

Suicide is an important public health problem and one of the leading causes of death among people aged 15 to 44 [1]. Globally, more than 800,000 people die due to suicide each year (http://www.who.int/mediacentre/news/releases/2014/suicide-prevention-report/en/). In Norway, 550 suicides were recorded in 2014; of these 443 used violent methods (http://statistikkbank.fhi.no/dar/) and 401 were males. Suicide attempt is found to be 10–15 times more frequent than suicide, and is considered the most predictive clinical risk factor for subsequent suicide [2–5].

A study from Sweden demonstrated that the risk of suicide was particularly high among those who attempted suicide by violent methods such as hanging, drowning, jumping from height or using firearms [6]. The relative risk for completed suicide was six times greater after an attempt by hanging and four times greater after an attempt by drowning than after an attempt by deliberate self-poisoning (DSP), which is the most common method of suicide attempt in Norway (https://www.fhi.no/nettpub/hin/helse-og-sykdom/selvmord-og-selvmordsforsok-i-norge/). The researchers also found that the short-term risk was particularly high shortly after an attempt by hanging, and that the same method often was used for both the attempted suicide and the successful suicide [6]. In addition, a British study showed that patients who had made suicide attempts by hanging had higher suicide intent and fewer used alcohol compared with patients who had used DSP [7]. Patients using violent methods like jumping have been studied, and Lindqvist et al. concluded that 64% had been treated in psychiatric clinic [8]. Surviving patients are at risk for repeated suicide attempts, often by using more violent and fatal methods [9, 10].

In Norway, only patients admitted to somatic hospital after suicide attempt with DSP have been studied. These patients have been extensively studied in comprehensive projects since 1980, e.g. covering the whole city of Oslo. Results show a high repetition rate, and the overall mortality 20 years after the suicide attempt was 45% for males and 30% for females [11]. Suicide was the mode of death in 7.1%. The standardized mortality ratios (SMR) were 4.6 for mortality and 26.7 for suicide. Of those who committed suicide, 40% used violent methods, demonstrating the serious prognosis after a suicide attempt.

Background, psychiatric and somatic conditions before and during the hospital stay and other precipitating factors in patients who make suicide attempts using violent methods in Norway, accordingly, is unknown. In search for studies that compare patients who commit suicide attempt by violent methods and patients with DSP, no previous studies were found. The mortality after using violent methods is high, as most suicide deaths in Norway are caused by violent methods, whereas most patients with suicide attempts use DSP. Knowledge about risk factors for a specific method of suicide attempt or suicide is important for evaluation of suicide risk in clinical settings in order to prevent future attempt.

The main objective of this study was to compare demographic data (age, gender, marital status, education and employment status), previous psychological health and psychiatric treatment, condition during stay (severity of injury, length of intensive care treatment, respiratory treatment and length of stay) in patients admitted to somatic hospital after attempted suicide using violent methods (hanging, drowning, jumping from heights, using firearms, cutting, or others) or DSP. The aim of this paper was to compare suicide attempt by violent method with suicide attempt by poisoning. By describing the patients we wanted to give an answer to our hypothesis, that patients who attempted suicide by violent methods were more often men and had a poorer psychiatric health before the attempt.

## Methods

This was a prospective cohort multicenter study. The following hospital trusts in Norway accepted the invitations to participate: Innlandet Hospital Trust, University Hospital of Northern Norway, Haukeland University Hospital (Bergen), Stavanger University Hospital, Akershus University Hospital and Tromsø University Hospital. Oslo University Hospital (OUS) is the Trauma center for south and eastern part of Norway and was the study’s administrative center. Patients hospitalized to somatic department after suicide attempt with violent methods included cutting, jumping from heights, hanging, shooting, drowning or others (like car accident on purpose, fire or jumping in front of train) were screened for inclusion. Patients were enrolled from December 2010 to April 2015. The study team visited all the hospitals that participated. This paper provides data from the baseline whereas 1 year follow-up data will be provided later.

### Patients

**Fig. 1:**
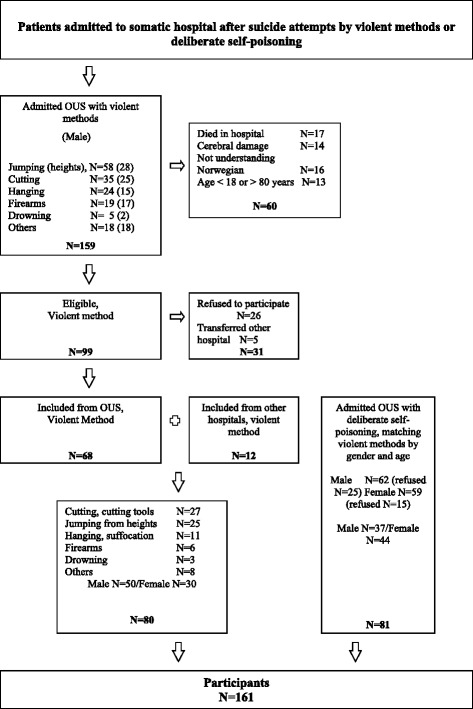
Flow chart

The inclusion criteria were: Patients aged 18–80 years who were admitted to somatic acute department after a suicide attempt. Because of the written forms and questionnaire, patients who did not understand oral and written Norwegian, who were mentally retarded, psychotic or did not have a permanent address were excluded. Suicide attempters classified as single wrist cut and other injury that did not cause any hospitalization were not included. Patients admitted for DSP were included after a patient using violent method had been included, in order to match according to age and gender. The patients were informed about the aim of the study, that it was voluntary to participate and that they at any time could withdraw their consent from the study without any consequence for the further treatment. The patients were invited to participate as soon as they were awake and able to consent and participate. When informed consent was obtained, the patients filled out the first questionnaire. If the patient needed help during the interview, this was assisted; either to read questions, help to write if e.g. the patient had hand injury or just to be there for guidance. The flowchart (Fig. 1) shows all 159 patients who were registered admitted to OUS with suicide attempt by violent methods during the study period. The violent methods that were used were in descending order jumping from heights 58 (30 females), cutting and cutting tools 35 (10 females), hanging and suffocation 24 (nine females), firearms 19 (two females), others (like jumping in front of trains and cars, fire etc.) 18 (10 females) and five drowning (three females). In total, 60 patients were excluded from the study (17 died in the hospital, 14 suffered from severe cerebral injury, 16 did not speak or understand Norwegian and 13 were younger than 18 years or older than 80 years). Of the 99 eligible patients, 26 (31%) refused to participate and five were transferred to other hospitals. The number of patients included with violent method from Oslo University Hospital was 68, whereas 12 were included from other hospitals. After a patient hospitalized with violent method was included, the intention was to include two patients admitted for DSP matched according to age and gender. The intention to include twice as many patients with poisoning was to get more statistical power. We did not succeed in getting that number of patients with DSP. We consider, however, the 81 included patients with DSP to be representative. Accordingly, it is unlikely that the main findings would have changed if the number of patients with DPS had been doubled, except possibly more statistically significant differences between groups.

### Measurements

The self-report questionnaire contained the following demographic variables: Gender, age, marital status, living conditions, educational- and employment status and current somatic and psychiatric condition, former DSP, previous deliberate self-harm and suicide attempt, hospitalization in psychiatric hospital or district psychiatric center (outpatient treatment) and attending GP. The answer categories were; “never”, “once”, “twice till three” or “four times or more”. Further questions about mental and somatic health, use of health care services, previous suicide attempts and hospitalization, suicide thoughts, self-harm episodes and thoughts when attending health care services and hospitals were added and translated into Norwegian from validated questionnaires [12, 13]. Finally, a question where the patients self-reported about the main cause to this hospitalization was included. The answer alternatives for this question were; “I wanted to die”, “escape from problems”, “affect relationships to someone”, “accident/intoxication”, “don’t remember”, “don’t want to state the reason” and “others”. The Karnofsky Performance Score runs from 100 to 0, where 100 is “perfect” health and 0 is “death”. The Karnofsky Performance Score was self-reported during the interview (we used range between 50 and 100) and described the patient’s physical health before hospitalization. The Karnofsky Scale Index classifies the patient’s functional impairment. This can be used to compare the effects of different forms of treatment and assessing prognosis in individual patients [14].

**Fig. 2:**
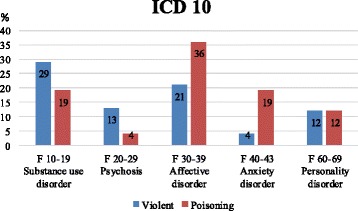
Psychiatric diagnoses according to method. Substance use disorder, *p*-value = ns. Psychosis, *p*-value< 0.05. Affective disorder, *p*-value< 0.03. Anxiety disorder, *p*-value< 0.002. Personality disorder, *p*-value = ns

In a separate form, we registered the following variables: Psychiatric diagnosis were classified in the following main diagnostic groups according to ICD-10 (http://apps.who.int/classifications/icd10/browse/2016/en): F10 – substance use disorder, F20 - psychosis, F30 - affective disorder, F40 - anxiety disorder, F60 – personality disorder (Fig. 2). We did not use a structured diagnostic interview, but the diagnoses were based on the clinical assessment of the psychiatric consultants, the medical doctors and information from medical and psychiatric records. Data on physical condition during hospitalization were obtained from the medical records. These were variables according to clinical data, respiratory treatment (yes, no), length of stay (days) and level of consciousness (Glasgow Coma Scale (GCS)). Generally, brain injury is classified as; severe GCS < 8–9, moderate 9–12 and minor > 13 [15].

Only at OUS, patients admitted with suicide attempt by violent methods had Injury Severity Score (ISS) and Simplified Acute Physiology Score (SAPS II) available. From the trauma register at OUS, ISS score was obtained. ISS measures the severity of injuries [16]. The ISS yields scores for overall severity of the injury from one to 75 and the scale is divided into four categories: 1–8 minor or moderate, 9–15 serious, 16–24 severe, and 25–75 critical injury [17]. The classification was based on injuries in chest, thorax and abdomen, head and extremities combined with GCS, blood pressure, pulse rate, respiratory frequency, intubation and need of trauma assistance at the time of admittance to hospital.

SAPS II is classifying the worst value of physiological variables within the first 24 h. The score obtains vitals (heart rate, blood pressure, and temperature), oxygenation (mechanical ventilation), renal function (diuresis) and blood sample and is a severity score and mortality estimation tool developed from a large sample of medical and surgical patients in North America and Europe. The SAPS II scores range from 0 (best) to 163 (worst) points. At 77 points, the mortality ratio reaches 90% [18].

### Statistics

Means and frequencies with SD or 95% Confidence Interval (CI) describe demographic and clinical data. We used the chi square test to compare differences between the groups on categorical variables. We used independent sample t- test to compare normally distributed variables. The level of statistical significance was set at *p* < 0.05. SPSS Second Edition (Version 23) was used to perform the statistical analyses [19].

### Ethics

The Norwegian Regional Ethics Committee and the Data Protection Officer at Oslo University Hospital approved this study (ref nr: REK: 2010/1487). The patients were informed about the aims and the study and signed a written consent. They were given an information leaflet containing the name and phone number of the study coordinator with the possibility of contact during the study.

## Results

During the study period, from 2010 to 15, 80 patients with violent methods and 81 patients with DSP were included. There were significantly more men (*n* = 50, 63%) among the 80 patients admitted for violent methods compared to patients with DSP (*n* = 38, 47%) (*p* < 0.05) (Table 1). There were no significant differences between the study groups on demographical characteristics. Few participants, 18% in the violent and 22% in the DSP group (*p* = 0.4), were working before the suicide attempt. Educational level was not significantly different according to college or university education in the violent group (29%) and the DSP group (36%) (*p* = 0.6).

**Table 1:** Demographic characteristics according to method

Number of patients, n (% or SD)	Violent methods *n* = 80	Deliberate self-poisoning *n* = 81	*p*-value
Gender			
Male	50 (63)	38 (47)	< 0.05
Female	30 (37)	43 (53)	
Age, years mean (SD)	42	42	
Male	43 (17)	45 (18)	ns (0.8)
Female	38 (14)	39 (16)	
Marital status			
Single	39 (48)	34 (42)	ns (0.5)
Married/cohabitant	23 (29)	27 (33)	
Separated	7 (9)	13 (16)	
Widow / widower	2 (3)	1 (1)	
In a relationship	9 (11)	6 (8)	
Living conditions			
Living alone	37 (46)	30 (37)	ns (0.7)
With husband/wife/cohabitant	24 (30)	28 (35)	
Alone with children	2 (3)	3 (4)	
With others	17 (21)	20 (24)	
Educational status			
Primary school	16 (20)	16 (20)	ns (0.6)
High school	41 (51)	36 (44)	
College / University	23 (29)	29 (36)	
Employment status			
Working	14 (18)	18 (22)	ns (0.4)
Unemployed	13 (16)	18 (22)	
Student	10 (13)	10 (13)	
Disability	25 (31)	11 (14)	
Retired	8 (10)	9 (11)	
Sick-leave	5 (6)	8 (10)	
Other	5 (6)	7 (9)	

Previous episodes of DSP and previous DSP were not significantly different between the groups (violent methods 74% vs. DSP 72%, *p* = 0.9) (Table 2). However, in the DSP group, more patients had an episode of self-harm the last month (40% vs 24%, *p* > 0.05 (Table 2) and a suicide attempt less than a week before this attempt (27% vs 15%, *p* < 0.05). Significantly more patients in the violent group reported this incident as a wish to die (62% vs. 55%, p < 0.05).

**Table 2:** Self reported previous health condition and use of health care services

Patients, n (%)	Violent methods N = 80	Deliberate self-poisoning N = 81	*p*-value
Karnofsky score^b^	93	96.7	*p* < 0.05
(0 min- 100 max)	(90.0–95.8)	(94.9–98.4)	
Previous somatic disease^a^	39 (49)	44 (54)	ns (0.5)
Previous episode of self-harm	59 (74)	58 (72)	Ns (0.9)
Poisoning - all	39 (49)	47 (58)	ns (0.2)
Once	12 (15)	23 (28)	
Twice or three	14 (18)	12 (15)	
More than three	13 (16)	12 (15)	
Cutting - all	30 (38)	29 (36)	ns (0.8)
Once	13 (16)	12 (15)	
Twice or three	6 (8)	5 (6)	
More than three	11 (14)	12 (15)	
Other- all	23 (29)	16 (20)	ns (0.2)
Once	14 (18)	8 (10)	
Twice or three	6 (7)	1 (1)	
More than three	3 (4)	7 (9)	
Time since previous self-harm			
< 1 month	19 (24)	32 (40)	*p* < 0.05
1–2 months	4 (5)	1 (1)	
3–5 months	10 (13)	0 (0)	
6–12 months	5 (9)	5 (6)	
1–4 year	9 (11)	9 (11)	
> 4 years	12 (15)	11 (14)	
Earlier episode of self-harm; − considered as suicide attempt	46 (58)	38 (47)	ns (0.4)
Last suicide attempt before the current hospitalization			
Less than a week	12 (15)	22 (27)	*p* < 0.05
< 1 month	4 (5)	5 (6)	
1–2 months	8 (10)	2 (3)	
3–12 months	9 (11)	2 (3)	
1–4 years	23 (29)	20 (25)	
Received psychiatric treatment before the current suicide attempt	38 (48)	44 (54)	ns (0.3)
Contact with GP	56 (70)	65 (80)	ns (0.1)
Frequency, last year			
Once	11 (14)	7 (9)	
Twice or three	15 (19)	18 (22)	
Four or five	11 (14)	16 (20)	
More than five times	19 (24)	24 (30)	
Last contact with GP before suicide attempt < 1 week	14 (18)	20 (25)	ns (0.5)
1–2 weeks	13 (16)	6 (7)	
2–4 weeks	15 (19)	14 (17)	
1–5 months	16 (20)	19 (24)	
6 months or more	17 (21)	18 (22)	
Reason for attending GP			
Physical disease	18 (23)	23 (28)	ns (0.2)
Psychiatric disorder	30 (38)	27 (33)	
Physical and psychiatric disease	24 (30)	29 (36)	
When you attended GP, were you planning to harm yourself?			
In a way	24 (30)	21 (26)	ns (0.6)
Yes, definitely	12 (15)	8 (10)	
Did you mention that you had thoughts about self-harming?			
Indicated it	16 (20)	12 (15)	ns (0.8)
Yes	9 (11)	9 (11)	
Earlier treatment in psychiatric ward?	37 (46)	32 (40)	ns (0.4)
Once	13 (16)	12 (15)	
2–3 times	10 (13)	12 (15)	
> 3 times	14 (18)	8 (10)	
Earlier treatment at district psychiatric center?	44 (55)	39 (48)	ns (0.4)
Once	16 (20)	11 (14)	
2–3 times	7 (9)	13 (16)	
> 3 times	21 (26)	15 (19)	
What do you see as the main cause of this hospital admission?			
I wanted to die	49 (62)	45 (55)	*p* < 0.05
escape from problems	12 (15)	23 (28)	
affect relationships to someone	1 (1)	4 (5)	
accident/intoxication	5 (6)	–	
don’t remember	5 (6)	2 (2)	
don’t want to state the reason	5 (6)	2 (2)	
others	3 (4)	5 (6)	

^a^Previous somatic disease; heart-, lung-, stomach/digestive-, diabetes/hormone disease, cancer, HIV or other somatic disease

^b^*GP* General practitioner

There were no significant differences in use of health care services in violent versus DSP group, as 48% vs 54% (*p* = 0.3) received psychiatric treatment, 70% vs 80% (*p* = 0.1) had contact with General Practitioner (GP) and earlier treatment at district psychiatric center 55% vs 48% (*p* = 0.04). Further, when attending the GP, no significant differences were seen regarding planning to harm themselves (violent group 45% vs DSP 36%, *p* = 0.6) and mentioned thoughts about self-harming (violent group 31% vs DSP 26%, *p* = 0.8). Patients admitted by violent methods reported lower Karnofsky score (93.0 vs 96.7, *p* < 0.05) than patients admitted with DSP.

About half of the patients in both groups were in psychiatric treatment at the time of the suicide attempt and had a previous medical record with an available F-diagnosis.

Patients with violent methods more often were diagnosed with psychosis than patients with DSP (13% vs 4%, p < 0.05). Patients admitted with DSP more often had anxiety disorders (19% vs 4%, *p* < 0.002) and affective disorders (36% vs 21%, *p* < 0.03) (Fig. 2).

During hospitalization, patients by violent methods were more frequently on mechanical ventilation than DSP (1.4 and 0.1 days, *p* < 0.001) and the average length of stay in hospital was 14.3 vs 2.3 days, and ICU stay was 4.9 vs. 0.6 days (both p < 0.001)). SAPS II and ISS scores describing somatic condition during stay in the intensive care were only provided in the violent method group included from OUS. Mean SAPS II and ISS score were 19.8 and 18.5 respectively (Table 3).

**Table 3:** Clinical data during hospitalization

Mean (CI)^a^	Violent methods N = 80	Deliberate Self-poisoning N = 81	*p*-value
Length of stay in hospital (days)	14.3 (8.3–20.3)	2.3 (1.6–3.1)	< 0.001
Length of stay in intensive care unit (days)	4.9 (2.9–7.0)	0.5 (0.2–1.0)	< 0.001
Mechanical ventilation (days)	1.4 (0.7–2.1)	0.1 (0.01–0.3)	< 0.001
Glasgow Coma Scale^b^	12.7 (11.8–13.6)	13.4 (12.7–14.1)	ns (0.2)
SAPS II mean	19.6 (16.2–23.3)	–	
ISS mean	21.2 (17.1–25.6)	–	

^a^Data presented as mean with 95% confidence interval (CI)

^b^Glasgow Coma Scale (3 min - 15 max)

Of all 159 patients admitted to OUS, 10.7% died in hospital. The methods used from the included patients in violent attempts (*n* = 80) were cutting (34%), jumping from heights (32%), hanging (14%), firearms (7%), drowning (4%) and others (10%) (Table 4). Patients using firearms had highest SAPS II score (mean 28 vs. hanging (lowest) mean 13) and highest mortality (21% vs. cutting (lowest) 3%), whereas patients admitted after jumping from heights were in the poorest condition after attending the hospital with the highest ISS score (mean 26 vs. cutting (lowest) 9). Most of these patients had severe physical injury. According to ISS, 19 patients had critical injury, 15 severe, seven with serious and 14 with minor or moderate injuries.

**Table 4:** Clinical data according to violent method

	Hanging	Drowning	Firearms	Cutting	Jumping	Others^a^
Admitted OUS with violent methods *n* = 159 Death^b^ n (%)	4 (2.5)	1 (0.6)	4 (2.5)	1 (0.6)	6 (3.8)	1 (0.6)
Included patients^c^ *n* = 80, (%)	11 (14%)	3 (4%)	6 (7%)	27 (34%)	25 (32%)	8 (10%)
Length of stay hospital (days) mean (min-max)	2.8 (0.5–8.6)	2.5 (0.2–6.0)	10.5 (3.2–25.8)	6.7 (0.4–43.5)	22.4 (0.7–119)	39.8 (0.4–143.7)
Length of stay Intensive care unit (days) mean (min-max)	1.7 (0.2–7.0)	2.5 (0.3–6.0)	9.0 (1.1–22.5)	2.0 (0.4–8.7)	8.7 (0.4–55)	1.8 (0.3–3.8)
Mechanical ventilation (days) mean (min-max)	0.6 (0.0–5.0)	1.3 (0.0–5.3)	2.0 (0.1–9.0)	0.4 (0.0–5.0)	2.5 (0.0–13.0)	0.8 (0.0–2.0)
Karnofsky score mean (min-max)	100^d^	90 (70–100)	100^d^	90.9 (60–100)	95.5 (70–100)	78,8 (60–100)
GCS^e^ mean (CI)	12.5 (9.9–15.0)	9.3 (5.6–24.3)	12.2 (7.5–16.9)	14.2 (13.2–15.1)	12.0 (10.1–13.9)	11.9 (7.3–16.5)
ISS^f^ range (min-max)	15 (1–27)	–	19 (5–25)	9 (1–26)	26 (1–57)	16 (10–29)
SAPS II^g^ range (min-max)	13 (2–23)	22 (18–27)	28 (14–52)	21 (7–37)	21 (4–46)	19 (9–28)

^a^Others: car, fire, acid, jumping in front of train/car

^b^Mortality in hospitalization. Data from patients admitted OUS with violent methods, *n* = 159

^c^Data from included patients with violent methods (*n* = 80) with percentages (CI) or range

^d^No variation

^e^*GCS* Glasgow Coma Scale (3 min, 15 max)

^f^*ISS* Injury Severity Score measures the severity of injuries

^g^*SAPS II* Simplified Acute Physiology Score is classifying the worst value of physiological variables within the first 24 h

## Discussion

The main findings in this study were that patients admitted to somatic hospital for suicide attempt with violent methods more often had a diagnosis of psychosis, the somatic conditions at admission were more serious, the mortality rate was higher and the hospital stay was longer than for patients with DSP. Cutting and jumping from heights were the most common methods, and use of firearms provided the most serious somatic condition and had highest in-hospital mortality.

Even though patients admitted with violent methods more often were diagnosed with psychosis, previous use of health care services, including psychiatric treatment, were similar in the two groups. This may be explained by the fact that anxiety and affective disorders were more frequent in the DSP group. Psychotic disorder were also found frequently (42%) in a previous study with suicide attempts by violent methods, where about half of the patients had never received treatment [20]. In our study, both groups have to a great extent been in contact with health care services and have a history of both self-harming and previous suicide attempts. This confirms previous knowledge about the high degree of repetition among suicide attempters [21]. Others have found that psychiatric admission was the highest risk for both suicide and mortality [22].

According to data from the general Norwegian population, two thirds of the population are employed (https://www.ssb.no/akumnd). In this study, only one out of five was employed before hospitalization. This is consistent with previous studies [23]. The low employment rate is somewhat in contrast to the educational level among the participating patients, which was similar to the national average (https://www.ssb.no/utdanning/statistikker/utniv). Higher education may therefore not be a protective factor when it comes to suicidal behavior (32% in our study were highly educated). In fact, the combination of high education and unemployment may be a risk factor because unemployment in educated groups may be perceived as worse. This study confirms that unemployment is associated with risk for suicide attempt [24–26].

Among all the patients admitted to OUS with violent methods, many had severe cerebral head injury and several patients died during hospital stay. The somatic condition was significantly more severe, and in-hospital mortality was significantly higher than for patients with DSP (none of the included patients died). Low in-hospital mortality in the DSP group is supported by previous studies of DSP (0.8%) [27]. Compared to an earlier study from the ICU department of trauma patients at OUS, the patients with violent method in our study had similar somatic injury compared to their study (mean ISS score 21.2 vs 23.1) [28]. The average length of ICU stay was also lower (4.9 vs 11.3 days) whereas in-hospital mortality was higher (10.7% vs 7.7%). There were differences in severity of the somatic injury (ISS) and in-hospital mortality according to the violent method used; patients with jumping had the most severe injury whereas patients who used firearms had highest mortality. Patients with the most severe condition died during the hospital stay and were excluded from our study. The ISS scores from these patients are therefore not included, and this may explain the lower mean score in our data compared with an average trauma population. Higher in-hospital mortality among patients admitted to OUS after suicide attempt with violent methods support that these patients are severely injured.

Methods used in suicide attempts and suicides are very different in different countries in the world. In this study, jumping from heights and cutting were the main violent method for suicide attempt for both genders. Compared to a Swedish study, cutting were the main methods in suicide attempters [6]. For suicide methods in Norway in 2015 were; hanging and suffocation 43%, DSP 20%, firearms 13%, drowning 6%, jumping 6%, cutting 2% and other 10% (http://statistikkbank.fhi.no/dar/). In British studies, jumping from heights accounted for 3–6% of all suicide attempts per year in United Kingdom [29, 30]. Worldwide, suicide by hanging is the most common method, with the highest prevalence in Eastern European countries [31], South Korea and Japan [32]. In many Asian countries and in Latin America, poisoning by pesticides is common, firearm is more frequently used in the United States and South America (most men) and among women poisoning by drugs most common in Canada, United Kingdom and some European countries [31]. Jumping from a high place is especially common in cities and urban societies such as Hong Kong and Singapore, where more than 40% of suicides are jumping from heights [33].

Demographics between the violent and the DSP groups were similar and similar to a Norwegian study of patients admitted to hospital after DSP [34]. As expected, most suicide attempts by violent methods were among men. This is in accordance with the suicide statistics in Norway [35] and from a Swedish study, where most men attempted suicide by violent methods [6]. In the DSP group, we found that more patients had an episode of self-harm the last month and a suicide attempt less than a week before the hospital admission. This complies with several previous studies of DSP which found that a history of a previous suicide attempt was the strongest predictor for both short- and long-term repeated suicide attempts [21, 24, 35].

Previous studies have found that patients who have jumped more often have been diagnosed with psychotic illness or borderline personality disorder than other suicide attempters [36]. Nielssen et al. found that 44% of the patients admitted after jumping were diagnosed with a psychotic illness and that 44% of them had not received treatment [37]. According to Cooper-Kazaz, several risk factors appear to be associated with a need for more intensive in-hospital treatment, such as male gender, method of suicide attempt and the existence of a psychiatric diagnosis [38]. They also found that patients were in need of hospitalization after a suicide attempt and required more intensive psychiatric treatment and follow up. In our study, most of the patients were in treatment by GPs and about half received psychiatric treatment before their suicide attempt and received diagnose before hospitalization.

### Strengths and limitation

There are no earlier studies were patients admitted to hospital after suicide attempt with violent methods are studied or compared with DSP in Norway. Most international studies have been based on mortality data and the distribution of different methods among suicide attempters is rare. The sparse literature in this topic, also outside Norway, makes comparisons with previous studies difficult. However, this makes the study results important, as they provide new knowledge about a condition with very high mortality rate in patients that often are young.

The intention of the study was to enroll 100 patients admitted to hospital with suicide attempt by violent method and subsequently 200 patients with suicide attempt by DSP, while the result was 80/81. A reason for this was slow recruitment at some sites. Further, there was insufficient access to men in the DSP group with matching age admitted to somatic hospital. From a large study of outpatient treatment of acute poisoning in Oslo, they found that 9% were DSP (of them 33% men) [39], and another large study of hospitalized patients from Oslo also shows that only one third of the DSP patients were male [40]. Different distribution of genders in DSP in outpatient clinic and hospitalized patients, even though the number of DSP are higher, including an age and gender match to the violent method, showed to be harder than expected. Some of the hospitals that should cooperate in the study were not dedicated to this task and most of them included very few patients. Only at OUS, the list of all patients admitted to the somatic hospital with violent method was provided.

How different organization of the services, different scientific culture and history of running studies contribute to these differences are unknown.

Even though the intention was to make the two groups similar according to age and gender, a much smaller number than first estimated turned out to be sufficient to find significant differences in physical sequelae, as we know that patients with poisoning have low prevalence of physical sequelae after DSP (0.6%) [41].

### Clinical implication

The results from this study provide new and relevant information for both somatic and psychiatric departments. As about half of the patients were in psychiatric treatment at the time of the suicide attempts, more effort must be provided to reduce the risk of suicide. Patients with previous suicide attempt and especially patients with psychosis will be in need of additional monitoring and treatment because violent methods with higher mortality rate were used more frequently in this group. Patients attending hospital after suicide attempt by violent methods are also in need of thorough somatic and psychiatric assessment and treatment during the hospital stay. Early during the hospitalization, it is important to determine the patient’s mental state in order to provide the best treatment during the stay and afterwards. It is difficult to predict outcome for these patients, but they all require multidisciplinary advanced treatment. It is challenging to capture those who do not express their suicidal thoughts without addressing these issues explicitly.

## Conclusion

Patients admitted with suicide attempt with violent methods more often had psychosis and less anxiety and affective disorders compared to patients admitted with DSP. Psychiatric treatment before the attempt and previous suicide attempt was not significantly different between the groups, and about half of the patients in both groups were in psychiatric treatment at the time of the suicide attempt. Patients with violent method had a more severe somatic injury and longer hospitalization than patients admitted for DSP. The in-hospital mortality in patients with violent methods was high. Close follow-up of patients after suicide attempt is needed, in particular for patients with psychosis and after suicide attempts with violent methods.
